# Lack of Association between Nuclear Factor Erythroid-Derived 2-Like 2 Promoter Gene Polymorphisms and Oxidative Stress Biomarkers in Amyotrophic Lateral Sclerosis Patients

**DOI:** 10.1155/2014/432626

**Published:** 2014-02-09

**Authors:** Annalisa LoGerfo, Lucia Chico, Loredana Borgia, Lucia Petrozzi, Anna Rocchi, Antonia D'Amelio, Cecilia Carlesi, Elena Caldarazzo Ienco, Michelangelo Mancuso, Gabriele Siciliano

**Affiliations:** ^1^Department of Clinical and Experimental Medicine, Neurological Clinic, University of Pisa, Via Savi 10, 56126 Pisa, Italy; ^2^Department of Clinical and Experimental Medicine, Neurological Clinic, University of Pisa, Via Roma 67, 56126 Pisa, Italy

## Abstract

Oxidative stress involvement has been strongly hypothesized among the possible pathogenic mechanisms of motor neuron degeneration in amyotrophic lateral sclerosis (ALS). The intracellular redox balance is finely modulated by numerous complex mechanisms critical for cellular functions, among which the nuclear factor erythroid-derived 2-like 2 (NFE2L2/Nrf2) pathways. We genotyped, in a cohort of ALS patients (*n* = 145) and healthy controls (*n* = 168), three SNPs in *Nrf2* gene promoter: −653 A/G, −651 G/A, and −617 C/A and evaluated, in a subset (*n* = 73) of patients, advanced oxidation protein products (AOPP), iron-reducing ability of plasma (FRAP), and plasma thiols (-SH) as oxidative damage peripheral biomarkers. *Nrf2* polymorphisms were not different among patients and controls. Increased levels of AOPP (*P* < 0.05) and decreased levels of FRAP (*P* < 0.001) have been observed in ALS patients compared with controls, but no difference in -SH values was found. Furthermore, no association was found between biochemical markers of redox balance and *Nrf2* polymorphisms. These data confirm an altered redox balance in ALS and indicate that, while being abnormally modified compared to controls, the oxidative stress biomarkers assessed in this study are independent from the −653 A/G, −651 G/A, and −617 C/A *Nrf2* SNPs in ALS patients.

## 1. Introduction

Amyotrophic lateral sclerosis (ALS) is an adult-onset neurodegenerative disease characterized by progressive degeneration of the anterior horn cells of the spinal cord [1] and cortical motor neurons [2]. Most of ALS cases (80–90%) are sporadic (sALS), and only 5–10% of cases have a family history (fALS). SALS and fALS are clinically indistinguishable and good evidence suggests that they basically share a common pathogenic mechanism that includes oxidative stress, excitotoxicity, and mitochondrial dysfunction [3, 4].

Mitochondria play central roles in cell's bioenergetics and apoptosis, and mitochondrial dysfunction appears to be involved in the pathogenesis of several neurodegenerative diseases, including motor neuron diseases [3]. Related to this, oxidative stress [5] and oxidative “free radical” damage are considered significant etiological factors in brain degeneration in ALS as demonstrated, for instance, by reported reactive oxygen species (ROS) tissue damage [6] and accumulation of oxidative damage to DNA, proteins, and lipids [4] within specific brain regions in this disease, even though it is still unclear whether oxidative stress is a primary cause of the pathogenesis of ALS or simply a result of the disease.

Multiple reports of oxidative damage in the spinal cord and in motor neurons of the motor cortex in *postmortem* tissues of both sALS and fALS cases are published revised in [7]. Among the mechanisms which can be involved in this contest, gene expression profiling of motor neurons that express the mutated form of *SOD1* shows a reduced regulation of some genes involved in antioxidant response, including the transcription factor Nuclear factor-erythroid 2- (NF-E2-) related factor 2 (*NFE2L2/Nrf2*) [8].

Nrf2 is a transcription factor “sensible” to free oxygen radicals' damage that is able to regulate the expression of many antioxidant pathway genes [9] and factors essential for neuronal survival [10].

In a recent paper, Holmström et al. showed how Nrf2 directly regulates cellular energy metabolism through modulating the availability of substrates for mitochondrial respiration [11]. He demonstrated that loss of Nrf2 leads to mitochondrial depolarisation and decreased ATP levels and impaired respiration; moreover, genetic activation of *Nrf2* increases the mitochondrial membrane potential and ATP levels, the rate of respiration, and the efficiency of oxidative phosphorylation. In vitro cultures of *Nrf2*-knockout cells revealed increased production of ATP in glycolysis and slower rate of NADH regeneration after inhibition of respiration than in their wild-type counterparts [11].

Desregulation of *Nrf2* expression and restoring balanced levels of redox-sensitive transcription factors and enzymes through *Nrf2* induction have been demonstrated in a number of mouse models of neurodegenerative disease. *Nrf2* gene has been demonstrated to be upregulated in reactive astrocytes in the spinal cord of symptomatic SOD1G93A animals [12, 13] and a reduction in *Nrf2* expression has been reported in neurons from primary motor cortex and spinal cord from ALS *postmortem* tissue samples [14]. A similar decrease in the mRNA encoding Nrf2 was observed in embryonic motor neurons isolated from hSOD1G93A rats [15].

Therefore, an overexpression of *Nrf2* in astrocytes has been demonstrated to be able to protect motor neurons, to delay onset, and to extend the median survival in SOD1G93A/GFAP-Nrf2 mice [16]. The same group surprisingly observed that an early and significant activation of the antioxidant response element (ARE) genetic system occurred in the distal muscles of mutant SOD mice, specifically in type I fibers that display an increase in *Nrf2* dependent transcription before symptoms onset in SOD1G93A mice [17].

Compilation of publically available SNPs and other genetic mutations shows that human *Nrf2* is highly polymorphic with a mutagenic frequency of 1 per every 72 bp. Functional at-risk alleles and haplotypes have been demonstrated in various human disorders, such as cancer, gastrointestinal and pulmonary disease, autoimmune disorders, and neurodegenerative diseases [18].

Functional polymorphisms in *Nrf2* that alters basal expression of Nrf2, or the ability of Nrf2 to translocate from the cytoplasm to nuclear binding sites, may cause redox alteration and production of oxidative stress damage. Proximal promoter SNPs in *Nrf2* regulatory regions and enhancer elements (such as the MZF1, AP1-like, and ARE-like sites) can affect transcription efficiency of the message, generating varying levels of mRNA and protein; it can also impact critical signal transduction pathways within the cell or tissue, finally playing key regulatory roles in various cellular responses after toxic and oxidant insults [19–21].

M. von Otter and colleagues found a region in *Nrf2* gene, including the promoter, which was clearly associated with risk of Parkinson's disease (PD) in two independent case-control materials. In particular, a haplotype including the fully functional variant of the promoter (GAGCAAAA) was associated with delayed age at onset in Swedish patients and reduced risk of PD in Polish patients [9]. In a recent paper of Ryu and collegues [22], phloroglucinol has been observed to be capable of attenuating motor functional deficits in an animal model of PD by enhancing Nrf2 activity. *Nrf2* haplotype alleles were also suggested as being associated with 2 years earlier age of Alzheimer's disease (AD) onset [23]. In a 2007 paper, Marzec and colleagues identified *Nrf2* as a susceptibility gene to hyperoxic lung injury in acute lung injury patients [24]. Three promoter polymorphisms (−617, −651, and −653 SNPs) were predicted to have functional significance, and one [−617 (C/A)] has been demonstrated to significantly affect basal *Nrf2* expression and function. They observed that these polymorphisms are predicted to affect Nrf2 ARE-like and MZF1 promoter binding sites [24].

The aim of this work has been to investigate molecular mechanisms that possibly underlie the regulation of cellular response against oxidative stress, through the evaluation of some oxidative damage plasmatic biomarkers and the analysis of −653 A/G, −651 G/A, and −617 C/A polymorphisms in *Nrf2* promoter gene.

## 2. Materials and Methods

### 2.1. Patients and Controls

We analysed a sample of patients and healthy controls with similar geographic origins (all patients and controls were Italians, from Tuscany) recruited at the Neurological Institute of Pisa University.

To genotype single nucleotide polymorphisms (SNPs) in *Nrf2* promoter gene, we analysed 145 sporadic ALS patients (88 males and 57 females, mean age ± SD 61.8 ± 11.4 years) and 168 unrelated healthy volunteers as controls, matched for age, sex, and ethnic background (84 males and 84 females, mean age ± SD 63.9 ± 10.7 years).

In a subset of 73 ALS patients (36/37 M/F, mean age ± SD 63.3 ± 10.8 years) and 68 controls (44/24 M/F, mean age ± SD 69.3 ± 9.2 years) we evaluated plasma oxidative stress markers.

All subjects gave their informed consent. The study was performed in accordance with the Declaration of Helsinki, last revision of Seoul (2008).

### 2.2. Methods

Blood samples were collected and centrifuged at 3000 rpm (600 g), within two hours of collection in order to obtain plasma which was stored at −20°C until analysis.

#### 2.2.1. Evaluation of Advanced Oxidation Protein Products (AOPP)

AOPP were determined according to Witko-Sarsat et al. [25]. Briefly, plasma was mixed with H_2_O, acetic acid, and potassium iodide. The absorbance was read spectrophotometrically at 340 nm and compared with a solution of chloramine T dissolved in the same buffer. The data were expressed as mmol/L of chloramine equivalents and related to plasma total protein, albumin, and immunoglobulin concentration.

#### 2.2.2. Evaluation of Iron-Reducing Ability of Plasma (FRAP)

FRAP was assessed according to Benzie et al. [26]. Briefly, the FRAP reagent (sodium-acetate, 2,4,6-tripyridyl-s-triazine in hydrochloric acid, and ferric chloride) prewarmed at 37°C was mixed with plasma; the absorbance was read after 3 min. at 593 nm. A calibration curve was established by substituting the sample with a solution of iron sulphate in hydrochloric acid.

#### 2.2.3. Evaluation of Plasmatic Total Thiols

The content of plasmatic total thiols was estimated by evaluation of the sulphydryl groups (-SH) present in the molecules, following the protocol described by Hu [27].

At the time of determination, 50 *μ*L of plasma is added to 150 *μ*L of a buffer consisting of Tris-EDTA, 10 *μ*L of 2,2-dithiobisnitrobenzoic acid (DTNB), and 800 *μ*L of absolute methanol. This is followed by an incubation time of 15 minutes at room temperature at the end of which the sample is centrifuged at 3000 g for 10 minutes. The absorbance of the supernatant was assessed at a wavelength of 412 nm and subtracting the value of a blank consisting of DTNB. The values of reduced glutathione are expressed in *μ*mol/L.

#### 2.2.4. Genotyping

The SNPs were analyzed using genomic DNA extracted from peripheral blood lymphocytes by automated sequencing. The genotyping protocol for the SNPs analysis was adapted from von Otter and coworkers [9, 23] and analyzed on an ABI PRISM 310 Automated Sequencer (Applied Biosystems, Forster City, CA, USA).

### 2.3. Statistical Analysis

To verify that allele frequencies were in Hardy-Weinberg equilibrium and to assess differences in the genotype and allele distributions between groups, we used the chi-square (*χ* ^2^) analysis using SPSS 11.0 for Windows operating system.

To evaluate the possible alteration of redox balance in ALS patients versus controls, we used unpaired two-tailed *t*-testing. The data were expressed as mean ± SD. The significance levels *α* used in this work are 5%, 1%, and 0.1%; the test has been called “statistically significant” for *P ≤ 5%.*

To assess differences in the genotype and allele distributions and to verify that allele frequencies were in Hardy-Weinberg equilibrium, we used the chi-square (*χ* ^2^) analysis. The distributions of genotypes and allele frequencies, related to the polymorphisms studied, are in agreement with the balance of Hardy-Weinberg.

## 3. Results

No significant difference was observed in genotype distributions between ALS cases and controls. The frequency of the variant allele G in −653 A/G polymorphism is lightly higher in patients than controls, but this difference was not statistically significant. Also no difference was detected when the polymorphism was stratified by gender (Table 1).

The frequency of the variant allele A in −651 G/A polymorphism is almost the same in patients and controls. Moreover, no difference was observed in genotype distributions between ALS cases and controls after stratification by gender (Table 2).

The frequency of the variant allele A in −617 C/A polymorphism in patients and controls is shown in Table 3.

There were no differences in allele frequencies or in genotype distributions between ALS cases and controls before and after stratification by gender.

**Figure 1:**
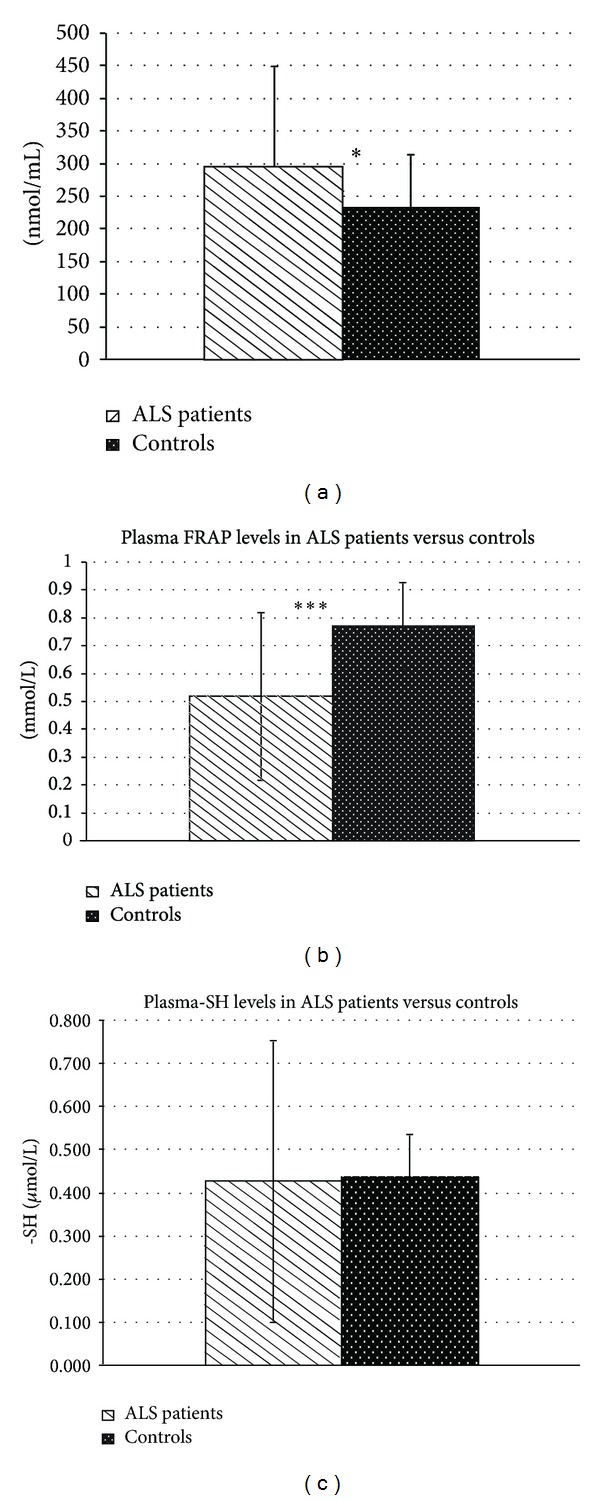
Variation of oxidative stress biomarkers in ALS patients compared to control group: (a) plasma AOPP levels (*P < 0.05), (b) plasma FRAP levels (***P < 0.001), and (c) plasmatic total thiols (*P* = n.s.).

Comparison between the selected patients (*n* = 73) and healthy controls (*n* = 68) subgroups has been shown in increased plasmatic AOPP levels (297.81 ± 151.0 nmol/mL versus 231.75 ± 81.92 nmol/mL; *P* < 0.05) (Figure 1(a)) and especially in reduced plasmatic FRAP (0.52 ± 0.3 mmol/L versus 0.77 ± 0.155 mmol/L, *P* < 0.001) (Figure 1(b)), while no difference was found in plasmatic total thiols levels (Figure 1(c)).

**Figure 2:**
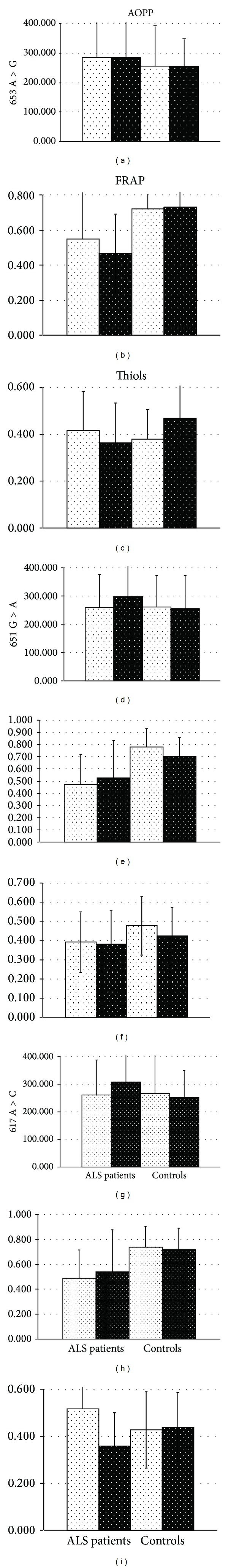
Association between *Nrf2* promoter gene SNPs and peripheral oxidative stress markers.

Furthermore, no association was found between the three polymorphisms of *Nrf2* promoter gene and peripheral oxidative damage markers, both in patients and in controls (Figure 2).

## 4. Discussion

Studies reporting the occurrence of altered redox balance in ALS patients, compared with healthy subjects [3, 28], highlight the crucial role of oxidative stress in motor neuron degeneration [29]. A bulk of evidence confirm the involvement of oxidative stress in neurodegenerative diseases and especially in ALS, although it is still widely debated whether or not oxidative stress is really involved in the pathogenesis of these disorders rather than to be merely an epiphenomenon. Therefore, despite the numerous studies carried out to better understand the role of oxidative stress in ALS onset, several points still remain unclear.

This study shows increased plasma levels of oxidized proteins in patients compared to controls, probably reflecting a state of altered redox equilibrium at a presumably very early stage of the disease, such as at the time of diagnosis, confirming a previous study performed by our group in 2007 [30]. Concordant with this, FRAP values show a decrease in plasma antioxidant capacity in patients compared to controls, in agreement with Keizman and colleagues [31] that found in ALS patients low serum levels of uric acid, which represents about 60% of the total value of FRAP. The reduction of serum uric acid has been demonstrated to be related to the rate of progression of the disease, further suggesting the possible role of oxidative stress in the induction and progression of the disease [31].

The values obtained from the analysis of these peripheral oxidative stress biomarkers appear to reflect a significant alteration of the redox profile in patients compared to controls, further supporting and confirming the hypothesis that oxidative stress plays an important role in the pathogenesis of ALS.

High ROS amounts cause macromolecular damages affecting proteins, lipids, and also nucleic acids. Protein oxidation can impair protein function, induce fragmentation, and promote promiscuous interactions that result in protein aggregation. The accumulation of intracellular protein aggregates may repress proteolysis, leading to cellular death by apoptosis. Moreover, oxidative stress also impacts translation and protein degradation, affecting protein expression levels in addition to changes at the mRNA level [32].

Based on recent data, altered control of gene expression seems to be a most relevant player in motor neurones diseases. Several studies addressing epigenetic modifications, transcriptomics, and proteomics of models and tissues from patients indicate that the overall pattern of gene expression is modified in motor neurones diseases, especially genes involved in defence responses, cytoskeletal dynamics, protein degradation system, and mitochondrial dysfunction in neurons [33, 34]. Several genetic factors involved in motor neurones diseases, such as *FUS*, *TARBP*, *ANG*, and *SMN* encode proteins with a role in RNA metabolism, supporting the concept that motor neurones diseases may be considered as “RNA dysmetabolisms” [35].

Nrf2 is a member of the cap “n” collar family of basic leucine zipper transcription factors that is able to “sense” free oxygen radicals' damage and regulate the expression of many antioxidant pathway genes in the so-called phase II response [9, 23] and factors essential for neuronal survival [10]. Nrf2, thus, appears to be an essential regulatory element in response to oxidant injury [36]; in fact Nrf2 is a crucial transcriptional factor that regulates the expression of many detoxifying enzymes such as NAD(P)H:quinine oxidoreductase, as well as classical antioxidant enzymes (catalase and superoxide dismutase) [37].

Under basal conditions, Nrf2 is mainly found sequestered in the cytoplasm by binding to Kelch-like erythroid-cell-derived protein with CNC homology (ECH)-associated protein 1 (Keap1) [38]. Keap1 is an actin-binding cytoplasmic protein that represses the transcriptional activation of Nrf2 because it sequesters the Nrf2 molecule from cell nucleus, preventing Nrf2 from activating target genes. Nrf2 is rapidly degraded by proteasomes through the interaction with Keap1 which interacts with Cullin 3, one of the components of ubiquitin ligase [39].

When the intracellular environment becomes toxic due to oxidative stress, Nrf2 can quickly translocate into the nucleus and elicit the antioxidant response [38]. In this cellular compartment, Nrf2 heterodimerizes with a small Maf protein and binds ARE, a regulatory enhancer region within gene promoters, leading to the transcriptional activation of phase II enzyme genes and antioxidant stress protein genes [14, 39]. Therefore, Keap1 and Nrf2 constitute a mechanism by which cells can sense free oxygen radicals' damage [14, 37].

As previously mentioned, Nrf2 involvement has been shown in neurodegenerative diseases such as AD, PD, Huntington disease [9, 22, 23, 40–48], and also in ALS models [12, 17]. In particular, three polymorphisms (−617, −651, and −653 SNPs) in the gene's promoter were predicted to have functional significance, and one [−617 (C/A)] has been demonstrated to affect significantly basal Nrf2 expression and function [9, 23]. Consistent with these hypotheses, we evaluate these three polymorphisms in a cohort of ALS patients, compared to healthy controls.

We did not observe correlations between the presence of each polymorphism and ALS, compared to healthy controls; nonetheless, the variant allele G in −653 A/G polymorphism was slightly higher in patients. According to Marzec and colleagues, this variant has been suggested to be capable of affecting *Nrf2* transcription [24], observing that the −538 to −727 region most likely contains DNA sequences required for high level Nrf2 promoter activity [24]. The negative effect of G variant of −653 polymorphism has been also described by Boettler et al. [49] that indicated reduced *Nrf2* gene transcription if the SNP at position −653 was present. This polymorphism has also been previously reported as associated with nephritis in childhood-onset systemic lupus erythematosus, although no significant association between susceptibility to SLE and Nrf2 polymorphisms was found [50]. This evidence and the presence of a higher frequency of this variant, albeit not significant, suggest a trend that should be further detected, possibly widening the cohort of patients.

Moreover we did not find any association between SNPs −653 A/G, −651 G/A, and −617 C/A in the *Nrf2* promoter gene and any of the redox biomarkers assessed in ALS patients. This may be considered in contrast with the concept that *Nrf2* pathway provides an effective “backup system” for cysteine-based redox regulation, predominantly via the transcriptional activation of GSH (the major thiol) synthetic enzymes [51]. In fact, the *de novo* synthesis of GSH is dependent on *Nrf2* that controls the GSH rate limiting enzyme *γ*-glutamyl cysteine synthetase (glutamate-cysteine ligase) with catalytic subunit C [52]. This is consistent with an improvement in redox GSH levels and neuron survival in AD models observed after Nrf2 activator treatment [52]. It can be hypothesized that these differences may be due to the different contest; in Satoh et al.'s study, in fact, biochemical assessments were performed on HT22 cells, a mouse hippocampal neuronal cell line [51], while, in Ghosh et al.'s study, on AD mouse neurons. It may be possible that such intracellular redox changes are not able to modify peripheral biomarkers values, at such an extent that they cannot be detectable enough by blood samples [52, 53].

## 5. Conclusions

Our results further support the theory that oxidative stress plays an important role in the neurodegenerative process. Although in our study we did not find any significant correlation between promoter's *Nrf2* SNPs and ALS and between these SNPs and the assessed peripheral oxidative stress biomarkers, the abovementioned bulk of literature, in showing *Nrf2* involvement in ALS, suggests to further investigate this field, possibly analyzing the entire *Nrf2* gene, as well as other molecular pathways correlated with it, as susceptibility factors in ALS.

As a whole, the demonstrated role of Nrf2-ARE pathway in oxidative stress modulation makes it an attractive therapeutic target for neuroprotection in ALS. Therapeutic tools acting on Nrf2 pathway seem to be able to modify disease course in some neurodegenerative disorders; for instance, intrahippocampal injection of a lentiviral vector expressing Nrf2 improved spatial learning in a mouse model of Alzheimer's disease [45].

Encouraging studies have been performed also in ALS models. The Nrf2/ARE activators CDDO ethylamide and CDDO 3-fluoroethylamide significantly attenuated weight loss, enhanced motor performance, and extended the survival of SOD1G93A mice [54], although these findings have not been confirmed in a subsequent study in which knocking out Nrf2 gene in SOD1G93A mice had only a modest impact on the course of disease [55].

S(+)-apomorphine, a nontoxic Nrf2 activating molecule, demonstrated not only CNS penetrance, Nrf2 induction, and significant attenuation of motor dysfunction in SOD1G93A transgenic mice, but also a reduced pathological oxidative stress and an improved survival following an oxidative insult in fibroblasts from ALS patients [56]. Lentiviral vectors expressing Nrf2 genes were tested in the ALS tissue culture model cells expressing the human SOD1G93A mutation. These cells overexpressing Nrf2 showed a significant decrease in endogenous oxidation stress levels. However, SOD1G93A mice did not experience any significant effect in survival, disease onset, or progression after administering, at a presymptomatic stage, intramuscular injections of adenoassociated virus serotype 6 expressing Nrf2 gene, even if that can be explained by the inefficient viral delivery [57].

Our observations highlight the importance of oxidative stress in ALS pathogenesis and confirm its involvement; the negative correlations in genotyping suggest wide studies on *Nrf2* gene role in this disease, in order to deepen knowledge into the pathogenic mechanisms underlying premature motor neurons' death and in view of finding new therapeutic strategies in ALS.

## Figures and Tables

**Table 1:** Distributions of genotypes and allele frequencies in SNP −653 A/G in ALS patients and in controls.

	ALS patients	Controls	*χ* ^2^ test	*P* value
Genotypes −653 A/G	*N*: 145	*N*: 168		
AA	71 (49%)	95 (56.5%)	—	—
AG	66 (45.5%)	62 (37%)	—	—
GG	8 (5.5%)	11 (6.5%)	—	—
AG + GG	74 (51%)	73 (43.4%)	1.8	0.18
Allele G frequency			0.8	0.36
Males	*N*: 88	*N*: 84		
AA	41 (46.7%)	44 (52.3%)	—	—
AG	45 (51.1%)	34 (40.5%)	—	—
GG	2 (2.2%)	6 (7.2%)	—	—
AG + GG	47 (53.3%)	40 (42.7%)	0.57	0.44
Allele G frequency			0.009	0.92
Females	*N*: 57	*N*: 84		
AA	30 (52.6%)	51 (60.7%)	—	—
AG	21 (36.8%)	28 (33.3%)	—	—
GG	6 (10.5%)	5 (6%)	—	—
AG + GG	27 (47.4%)	33 (39.3%)	0.9	0.34
Allele G frequency			1.5	0.21

**Table 2:** Distributions of genotypes and allele frequencies in SNP −651 G/A in ALS patients and in controls.

	sALS patients	Controls	*χ* ^2^ test	*P* value
Genotypes −651 G/A	*N*: 145	*N*: 168		
GG	105 (72.4%)	130 (77.4%)	—	—
GA	40 (27.6%)	35 (20.9%)	—	—
AA	0	3 (1.7%)	—	—
GA + AA	40 (27.6%)	38 (22.6%)	1.02	0.31
Allele A frequency			0.35	0.55
Males	*N*: 88	*N*: 84		
GG	66 (75%)	68 (80.9%)	—	—
GA	22 (25%)	16 (19.1%)	—	—
AA	0	0	—	—
GA + AA	22 (25%)	16 (19.1%)	0.88	0.34
Allele A frequency			0.77	0.37
Females	*N*: 57	*N*: 84		
GG	39 (68.4%)	62 (74%)	—	—
GA	18 (31.6%)	19 (22.4%)	—	—
AA	0	3 (3.6%)	—	—
GA + AA	18 (31.6%)	22 (26.2%)	0.48	0.47
Allele A frequency			0.95	0.32

**Table 3:** Distributions of genotypes and allele frequencies in SNP −617 C/A in ALS patients and in controls.

	sALS patients	Controls	*χ* ^2^ test	*P* value
Genotypes −617 C/A	*N*: 145	*N*: 168		
CC	109 (75.2%)	123 (73.2%)	—	—
CA	34 (23.4%)	41 (24.4%)	—	—
AA	2 (1.4%)	4 (2.4%)	—	—
CA + AA	36 (24.8%)	45 (26.8%)	0.09	0.7
Allele A frequency			0.28	0.59
Males	*N*: 88	*N*: 84		
CC	62 (70.4%)	59 (70.3%)	—	—
CA	24 (27.3%)	21 (25%)	—	—
AA	2 (2.3%)	4 (4.7%)	—	—
CA + AA	26 (29.6%)	25 (29.7%)	0.001	0.98
Allele A frequency			0.09	0.75
Females	*N*: 57	*N*: 84		
CC	47 (82.5%)	64 (76.2%)	—	—
CA	10 (17.5%)	20 (23.8%)	—	—
AA	0	0	—	—
CA + AA	10 (17.5%)	20 (23.8%)	0.5	0.47
Allele A frequency			0.7	0.4
